# A Formative Qualitative Study on the Acceptability of Deferred Consent in Adult Emergency Care Research in Malawi

**DOI:** 10.1177/1556264619865149

**Published:** 2019-08-08

**Authors:** Lucinda Manda-Taylor, Fanuel Meckson Bickton, Kate Gooding, Jamie Rylance

**Affiliations:** 1 University of Malawi, Blantyre, Malawi; 2 Malawi-Liverpool-Wellcome Trust Clinical Research Programme, Blantyre, Malawi; 3 Liverpool School of Tropical Medicine, UK

**Keywords:** emergency care research, deferred consent, acceptability, Malawi

## Abstract

Research in emergency medical care is challenging due to a limited therapeutic window for intervention, which may compromise informed consent. “Deferred consent” allows initiation of study procedures before full consent is recorded. We conducted a formative qualitative study exploring perspectives on deferred consent in Malawi among research ethics committee members, health care professionals, and lay representatives. Participants identified several advantages of deferred consent including scientific value and potential health benefits to the study subjects and wider population. Participants also had concerns, including regulatory barriers and the risk of abuse and malpractice. Conditions affecting acceptability are related to the role of proxies, the nature of the research, the availability of robust regulatory oversight, and the need for community engagement. Our findings show deferred consent would be acceptable in Malawi, provided that a clear case can be made to advance medical knowledge and that adequate regulatory and ethical protections are in place.

## Introduction

Medical interventions in emergency care can be critical to patient survival. Early treatment is intuitively sensible and demonstrated to improve survival in emergency conditions such as myocardial infarction, sepsis (severe infection), and stroke. However, evidence for specific interventions is limited by (a) the difficulties of performing research in a time-pressured environment and (b) a selection bias caused by only performing research on less-severely affected patients (Tu et al., 2004).

One constraint on emergency care research (ECR) is the complexity around obtaining full informed consent prior to a research intervention. Informed consent is understood to be a free choice made by an individual with “capacity” to do so, where capacity requires all of (a) an understanding information relevant to the decision, (b) the ability to retain the information, and (c) the ability to communicate the decision (Gupta, 2013). ECR represents an exceptional situation in which the mechanisms of the consent process may need to be modified, but the social contract between researcher and research subject must be respected to provide a safeguard against unethical research (Jansen et al., 2007).

The protection of human subjects with diminished autonomy or capacity presents ethical and practical challenges. Impairment of capacity may result from the effects of the illness itself, or the circumstances of emergency medical management, such as insufficient time to weigh decisions, and reduced clarity of thought in an inherently stressful environment (Jansen et al., 2007; Mason & Allmark, 2000). When patients lack capacity to consent for themselves, especially in emergencies, they are frequently treated by doctors without such consent, according to the patient’s best interests. However, research interventions are seen differently; there is “equipoise,” meaning that the balance of benefits and risks are uncertain and best interests are not known. An inability to garner evidence due to lack of informed consent leads to persistent uncertainty. As a solution, alternative approaches have been applied in both clinical and research practice, such as seeking consent from a proxy, waiver of consent, consent by an independent physician, and “deferred consent” (Jansen et al., 2007). Each approach has an impact on patient autonomy and, subsequently, the use of data and interventions in the absence of explicit consent from the patient.

International ethics guidelines for the use of deferred consent (DC) in research vary. The *International Ethical Guidelines for Biomedical Research Involving Human Subjects* by the Council for International Organizations of Medical Sciences (CIOMS; 2016) and UNESCO’s Universal Declaration on Bioethics and Human Rights (n.d.) state that any waiver or delayed consent should be a rare exception and subject to full ethics committee review and must bring direct benefit to the participant. Australian regulatory authorities accept ECR, if it is approved by an ethics committee as having an appropriate risk–benefit ratio and if there is no obvious reason why the participant would refuse consent had they been capable of consenting (National Health and Medical Research Council, the Australian Research Council and Universities Australia, 2007). In the United States, the Food and Drug Administration allows exemptions for informed consent for life-threatening illness where (a) evidence on interventions is lacking; (b) neither informed nor proxy consent are possible as a result of the patient’s condition; (c) participation might offer direct patient benefit because immediate intervention is necessary, there is support from animal or preclinical studies for potential benefit, and the risks are “reasonable in relation to what is known about the medical condition”; and (d) additional protections are in place. Such protections include community consultation, public disclosure of the research plan and its outcome, and an independent data monitoring committee (all of which would be good practice in interventional research) (U.S. Food & Drug Administration, 2011). Similarly, the U.S. Department of Health and Human Services (DHHS; 2013) permits consideration of research without informed consent in emergency situations if five stringent criteria are met: acutely life-threatening condition, current treatments are untested or unsatisfactory, lack of capacity is due to the acute condition, prospective consent from a proxy would introduce life-threatening delay, and the subject might directly benefit from participation. In Europe, legislation aims to prevent exposure of patients to unvalidated clinical practice (nonmaleficence) and to improve the prognosis of disorders requiring emergent treatment (beneficence) (Mentzelopoulos, Mantzanas, van Belle, & Nichol, 2015). Participants can be enrolled into studies without full written consent, premised on the need to increase evidence-based improvements in emergency care, and researchers are required to seek full consent at a later time (Mentzelopoulos et al., 2015). By stipulating that there should be the possibility of direct patient benefit, both the U.S. and European guidelines remain consistent with the Nuremberg Code, that “science and society’s interests in medical progress may never overrule or outweigh the interests of participants potential burden and risks for patients” (Jansen et al., 2007).

Within the sub-Saharan region, Section 2.3.9 of the South African Good Clinical Practice expressly states that ECR is allowable provided that clear reasons to justify the initiation of ECR without consent are given (Department of Health, 2006). In Malawi, current ethics guidelines do not specifically address situations of ECR when patients lack capacity to consent. However, there are relevant principles in the existing regulatory framework. National regulatory guidelines stipulate that written informed consent must be obtained from each research participant who is legally, mentally, and physically able. This is supported by the constitutional edict: “No person shall be subjected to medical or scientific experimentation without his or her consent” (Constitution of the Republic of Malawi, 1994). For those unable to fulfill the above conditions, consent is sought from parents (in the case of minors), legal guardians, or legally authorized representatives (National Commission for Science and Technology, 2011; Toto, 2016). The waiver of informed consent is regarded as exceptional, and must in all cases be approved by an ethical review committee (National Commission for Science and Technology, 2011; Toto, 2016).

The lack of uniformity, direct and clear ethical guidance creates ambiguity for investigators about appropriate consent procedures for ECR and can potentially discourage needed research or lead to inconsistent and potentially unacceptable consent approaches. Given the large need for ECR in low-to-middle-income countries (LMICs) due to the high burden of disease, research on the acceptability and applicability of deferred or delayed consent is needed to support clearer guidelines. There is currently limited evidence on acceptability of deferred consent in sub-Saharan Africa (Furyk et al., 2018). For pediatric populations, acceptability has been demonstrated in a multicenter trial in the region (Maitland, Molyneux, Boga, Kigali, & Lang, 2011). However, for adult populations, the views of stakeholders have not been widely sought or published. We performed a formative qualitative study to examine the acceptability of deferred consent for research studies of emergency care in Malawi.

## Method

### Participants and Data Collection

We conducted a qualitative study, involving interviews with five REC (research ethics committee) members and six health care providers, and one focus group that comprises 12 local community members. REC members and health care providers are purposively sampled based on their knowledge and experience of consent in research and experience of emergency care settings. We included representatives from the National Health Sciences Research Committee (NHSRC) and the College of Medicine Research and Ethics Committee, and both doctors and nurses familiar with emergency care in a tertiary referral hospital in Blantyre. The community focus group comprised members of the Malawi-Liverpool-Wellcome Trust (MLW) Blantyre Community Advisory Group, which includes people familiar with local community views (e.g., community-based organization [CBO] leaders and teachers). In-depth interviews (IDIs) and focus group discussions (FGDs) were conducted between June 2017 and May 2018. We used separate semistructured topic guides (Supplemental Appendices 1, 2, and 3), with questions related to experiences with deferred consent in ECR involving adults, the perceived benefits and risks of deferred consent, the acceptability of deferred and proxy consent, and the regulatory capacity and framework required to provide ethical oversight and governance to such research.

We employed a purposive sampling strategy in an iterative process to allow for the inclusion of a range of stakeholder views to reflect the opinions of people with differing expertise and knowledge to establish saturation in each group. To ascertain whether participants had some exposure to the concept of deferred consent, a definition of deferred consent was provided. In addition, vignettes of examples on types of researchers were presented to facilitate further discussion on the use of deferred consent in ECR (Supplemental Appendices 1, 2, and 3).

Although the sample is small, we found that similar themes were emerging by the time of the later interviews and judged that saturation had been reached. The IDIs were conducted in English and the FGD in *Chichewa* (the local language). Data were audio-recorded and transcribed, and the FGD transcript was translated into English. Transcripts were reviewed for completeness and accuracy against the recorded interviews.

### Data Analysis

Data analysis was ongoing throughout fieldwork to allow inclusion of any emerging issues in later interviews and support decisions on further data collection. Data were analyzed thematically. All authors familiarized themselves with the whole data set, coded two to four transcripts each and developed a common coding framework through group discussion. All transcripts were coded by at least two individuals, using NVivo 11 software (QSR International, Victoria, Australia). Key themes were identified and discussed by all authors and reviewed for referential adequacy by returning to the raw data (Nowell, Norris, White, & Moules, 2017).

### Ethical Approval

The University of Malawi’s College of Medicine Research and Ethics Committee (P.04/17/2162) granted ethical approval. Permission to collect data in Queen Elizabeth Central Hospital was provided by hospital director. Written informed consent was obtained from all participants during data collection. Literate participants provided a signature on the consent form and illiterate participants provided a thumbprint. Interviews were conducted in a private space, and participants assured that their details would be omitted from transcripts and no personal details would be divulged to ensure confidentiality. Finally, participants were informed that their involvement in the research was voluntary and that withdrawal was permitted at any time and without personal consequence.

## Results

All REC members were male; the health care workers (HCWs) group consisted of two males (one medical doctor and one nurse) and four females (two medical doctors and two nurses) and the CAG (community advisory group) consisted of seven males and five females (Table 1).

**Table 1.** Demographic Attributes of the Participants.

Participant group	Sex		Total
	Male	Female	
REC members	5	0	5
HCWs	2	4	6
CAG members	7	5	12

*Note.* REC = research ethics committee; CAG = community advisory groups; HCW = health care worker.

Key themes identified were related to experience with deferred consent, perceived benefits of deferred consent, perceived risks of deferred consent, and conditions affecting the acceptability of deferred consent. We address each of these themes in turn below.

### Experience With Deferred Consent

There was limited experience with deferred consent in the research context. All REC members stated that they had never reviewed research protocols proposing use of deferred consent in Malawi, and no community members had direct experience or knowledge of deferred consent. HCWs had experience with the use of deferred consent for incapacitated patients, but rather than for clinical care patients in research:

. . . consent is usually given by the relations but working in accidents and emergency center there are times when you go ahead and do procedures because the patient is incapacitated; either they cannot speak or airway is blocked. So we go on to intervene because whatever we are doing, we are doing at the patient’s best interests. So I think in medical care, there are times when informed consent can be overridden based on the patient’s situation, but not necessarily for research purposes. (HCW IDI 03)

This lack of experience reflects the situation in Malawi, where deferred consent has not yet been employed for ECR.

### Perceived Benefits of Deferred Consent

Views on the perceived benefits of deferred consent varied between REC members, HCWs, and CAG members, but most participants thought deferred consent was a beneficial approach to employ for ECR. Key benefits identified by participants were related to the scientific value, improvements for population health, and health benefits for individual participants. For example, in relation to scientific value and population benefit, participants described the need for ECR to improve medical care in such situations:

. . . obviously if you never studied things like this you would never move forward in medical advances. (REC KII 05)

I think for interventions that would require or that need to be, you know, trialed or investigated in the context of very sick patients . . . it would be appropriate to test those interventions through use of deferred consent. (REC KII 04)

Another benefit, mentioned particularly by CAG members, was related to the individual health benefits that could potentially be derived from ECR:

This research is good because when the person is sick and falls unconscious, we don’t know what has caused this. But if research is done on them, the underlying cause can be identified. (CAG FGD P 01)

One REC member was of the view that deferred consent is permissible in ECR, if it is to contain an epidemic, so as to improve the life and health of the individual and by extension the community and general population:

. . . let’s say you are conducting a trial of a serious rare disease and maybe there is no treatment and you have possible treatment. Let’s say Ebola . . . I would think in that situation, because it’s about saving the patient’s life. If you start looking for legally authorized representative maybe that is going to take time .So in that circumstance I would think a deferred consent would be justifiable. (REC KII 01)

This view was echoed by an HCW who noted the importance of rapid intervention:

So if we delay because we are waiting for some form of consent, then that therapy will not have the positive effect that we are hoping it to have. (HCW IDI 01)

Other participants expressed that deferred consent was justifiable and beneficial, if the research would improve clinical care:

Deferred consent should be done for studies that have pertinent population importance. . . it shouldn’t just be done just because people want to answer some intellectual question that will not be relevant to clinical practice or make a significant difference to the patients, to the population. (HCW IDI 02)

### Perceived Concerns about Deferred Consent

Participants raised several concerns about conducting research that applies deferred consent. One concern, mentioned by an REC member, was related to how the use of deferred consent in research was against the law in Malawi:

. . . the constitution gives the caregiver power to authorize research on a person but the clinicians are not obliged to authorize consent on behalf of patients. No way is that allowed. . . It doesn’t authorize the researcher or clinicians to authorize any research intervention on a patient without any consent of the two, either the individual himself or the relative. (REC KII 02)

Another concern that was mentioned by an HCW and other participants in the study related to the risk of abuse and malpractice, with potential neglect of patients’ rights is as follows:

In Malawi, it’s easy to be abused. (HCW IDI 02)

This risk of abuse and malpractice is, in part, a result of the medical condition of the patient and the fact that it could be easy to get away with recruiting people who are very sick:

How do you take care of the situation where people just want to abuse as the patient is too sick? So how do you take care of that situation if people just take advantage, saying “the patient was too sick so I just deferred the consent so I still recruited them.” (REC KII 01)

. . . the patient doesn’t know what the researchers are taking from them such as fluids or they don’t know anything the researchers are going to do on their body. So because the patient is unable to consent, the researchers can do anything they like on the patient. This is its badness. (CAG FGD P11)

### Conditions Affecting the Acceptability of Deferred Consent

Participants discussed conditions that could make deferred consent feasible and acceptable to ethics committees, HCWs, and communities in Malawi. Several issues were described, including the nature of the research, scientific and public health justification, appropriate proxy consent, adequate regulatory oversight, and robust community engagement.

#### Nature of research

The study type (observational or interventional) and the level of risk were thought to be determinants of the limits of applying a deferred consent approach. Some health workers suggested that deferred consent would be more appropriate with lower risk studies:

. . . If we are looking at something that’s minimally harmful like, for instance, looking at the use of lactate . . . to direct therapy is potentially beneficial, for instance. But if we are looking at a new medication. . . it might be tricky to use deferred consent in that case because if we are not fully aware of the potential risks. (HCW IDI 01)

Considering this issue of risk, this health worker described observational studies as more feasible, but considered that intervention research may be possible providing risk levels are acceptable:

Observational is easier [uhm] because there is less harm. . . compared to interventional. So, for interventional. . . how much do we know about its possible risks and the possible benefit? (HCW IDI 01)

#### Scientific justification

Scientific justification for the use of deferred consent and potential contribution of the research to individual health was clear conditions affecting the acceptability of deferred consent, and these should be explicit:

There must be proper justification on why you choose deferred and not normal informed consent or why you don’t want proxy informed consent. (REC KII 02)

Similarly, participants described the need for research interventions to have the potential to save the life of patients and the relevance of the research to the study population:

If you did a trial or a study where there is no treatment and you are trying out product that may potentially save the patient’s life that the only case you could use that. (REC KII 01)

Deferred consent should be done for studies that have pertinent population importance. It should not just be done out of interest of a researcher and they just want to do them. (HCW IDI 02)

#### Getting proxy consent

For many respondents, getting proxy consent was a pragmatic approach, with reasons being related mainly to culture, protection of the confidentiality of the patient’s information, and protection of the researcher from litigation. Culturally, proxy consent was an important part of respecting established societal structures:

. . . in the Malawian context . . . no matter how important it may be, the guardian should be considered. Only when maybe the patient is unknown, yes, but if the guardians are there get the consent from the guardians. (HCW IDI 04)

Although proxies were cited as important, respondents also revealed several challenges engaging them. One challenge related to poor communication, particularly, in the context of low health literacy and where death may be blamed on the decisions of researchers:

This time the patient has died, it’s the guardian. How are you going to explain that, if the patient has died? But maybe because many people may not know what you (healthcare workers) do at the hospital or what treatment you give etc, depending on the level of education. (HCW IDI 04)

Yes, I mean if the patient is in coma and deferred consent is happening, if the patient dies and if the relatives knew that the deceased was being researched, the relatives will accuse the doctors for being responsible for the death. (CAG FGD P10)

Another challenge with ECR, as one participant observed, related to the risk of delayed treatment to the patient in circumstances where the proxy is not readily available is,

The disadvantage of this research is that it can take a very long time; because if the patient dies, and if the guardian of the deceased is too far away, the researchers will be waiting for the guardian to arrive, which can take more time. (CAG FGD P03)

However, most community representatives were of the view that in such circumstances, the doctors and/or researchers should just proceed without obtaining proxy consent:

. . . let the researchers do their job. Because if you wait for my relatives to come first all the way from Chileka or Lilongwe, the patient can die. (CAG FGD P 07)

There were different views on who was best placed to act as a proxy. Many participants saw relatives as the appropriate proxy and provided examples of which relatives would be appropriate:

. . . I think every household has got a bread earner. So the bread earner stands a chance to give that. If not, as Malawians we have structures; the uncle is the most powerful individual in the family. So give him a stance to give consent. (HCW IDI 03)

However, views also varied on which person was the recognized representative with the cultural powers to act as a proxy:

People who are typically from the villages and whatever even the husband may not have the mandate; they will say *mwinimbumba* [the family’s genealogy owner]. (HCW IDI 04)

Community members were generally in favor of HCWs acting as proxies because they appeared to place more trust in HCWs and researchers to do their job and act in the best interests of the patient as described:

I agree that a proxy consent should be taken from an independent doctor because the doctor’s consent is more likely to be rational/well-informed than the guardian who just responds out of ignorance. So, yes, it is important that an independent doctor can give proxy consent. (CAG FGD P 05)

Some saw use of doctors as particularly appropriate when relatives or guardians were not available:

An independent doctor should be involved because sometimes there may be bad blood between a patient and guardian and the patient may come to [the hospital] with no guardian (because of the bad blood) and be in a critical condition. Doctors should be involved in the decision making about the patient’s management and it doesn’t make sense to leave the patient unmanaged just because there is no guardian around. An independent doctor should come in to let the patient be researched. (CAG FGD P 10)

However, in contrast, all the REC members felt that proxy consent by independent doctors was not appropriate and suggested that proxies should be caregivers or patient advocates instead. These concerns are related to both the legal framework and risks that decision making would be influenced by social networks and not be independent:

I think that again it—[consent by other doctors] would be difficult. . . This is an old boys network, you could just call up your mate to come and give consent. I think you would need a definitely independent . . . we actually have patient advocates. People whose job was to protect patients. . . You would get the advocates to come along and ask them as an independent person. (REC KII 05)

HCW views varied. Some HCWs preferred use of close relatives as proxies and had concerns about use of doctors, related to both paternalism, and the risk of regrets if the patient does not survive:

. . . if one gives that deferred consent and it doesn’t work well maybe the patient. . . something happens—prognosis is poor or the patient dies—it’s a “Oh, I wish I would not have done that.” (HCW IDI 04)

#### Adequate regulatory capacity

Issues of capacity building and legal permissibility were emphasized in creating a robust regulatory environment for all the players involved in reviewing, approving, monitoring, facilitating, and conducting research. Key suggestions included training and adequate professional expertise for ethics committees:

the level appreciation of bioethical principles amongst most reviewers in Malawi is minimal . . . it would require special, I don’t know what word to use, maybe training or sensitization of the members of the ethics committees about the sensitivity surrounding issues of deferred consent. (REC KII 02)

. . . when they are reviewing the protocols, they have someone with expertise. . . to properly evaluate the risks and benefits of whatever intervention is being investigated, and whether indeed the outcome of the research has potential for benefit, either to individual or society. (HCW IDI 01)

But nonprofessional input was also valued in reviewing and monitoring studies:

A layman’s board on the ethics committee to get the society’s point of view. . . to have a patient advocacy group that you could refer to. . . To check that they aren’t playing fast and loose with the consent that they are entering the patient but they are definitely getting the deferred consent in some way, shape or form. (REC KII 05)

#### Community engagement

Community engagement to improve understanding of deferred consent was also considered necessary. This included raising awareness through dialogues with community members to explain the potential risks and benefits of participating in ECR using interventions whose safety or efficacy remains largely unknown:

I think mostly at the community level. Maybe there is need for awareness and civic education on the importance of research. (HCW IDI 05)

Limitations of public engagement were recognized, but improving awareness in the communities at large was considered important:

if you are looking at illiterate population then it’s difficult for. . . it’s very easy for such a population to be duped into participating, accepting research that’s harmful to the society. On the other hand, it’s also easy for such communities to just refuse even beneficial research. So I think (1) literacy levels have to improve, and then (2) research groups have to have the community component where the communities are well informed about what is happening. (HCW IDI 01)

### To Use or Not Use Data When Patients Die Before Providing Consent

One particularly challenging situation in ECR relates to use of patient data in the event that the patient dies before recovering sufficiently to be asked for consent. When asked specifically, many respondents felt the use of patient data would be permissible if a patient died to avoid selection bias in recruitment of patients and to minimize biases in presentation of study results.

Yes, it matters a lot. Because the bias will still come to say like you only recruited this type of patient and you left the majority like this type of patient. So that will also have effect on the end result of the study. And I have observed that like most of the patients who are very sick, even the researchers they don’t approach them; they say this one is already sick, will die soon. . . So I have seen a lot of patients who could have benefited or could have contributed to most of the studies but they were left out because they couldn’t give consent. . . . (HCW IDI 06)

. . . it could be unethical not to use them for a simple reason that you would bias your results. If you excluded everyone that died because you haven’t been able to get consent you are actually going to influence your results, aren’t you? You are going to select the ones that are going to die so then you try and publish it saying whatever it is that your hypothesis is. You are actually publishing false data. That’s prejudice by the fact that you are excluding dead people. (REC KII 05)

Others were cautious and felt that proxy assent must be sought for ongoing use of the data collected:

No. You list them that they died before consent. Because if you use that data it means you have done without any consent. (HCW IDI 05)

I would think it is unethical to do so because of the consideration that the interest of the patient must override the interest of science in clinical, new research. . . Somebody who has died, I think it’s a bit of disrespect to the cultural system. (REC KII 03)

. . . when a person has died the patient himself cannot give consent it’s only the caregivers who can give consent . . . once you die you lose your autonomy and that autonomy is passed on, the authorization power is given to either the parent or recognized caregiver. (REC KII 02)

Safeguards, such as ensuring any collected data are anonymized after the patient dies, were suggested as a way of adding extra protection, maintaining privacy, and upholding confidentiality:

So I think it will depend on the study whether names will be removed—coding will be done—to safeguard the patient’s interests because if the patient dies, it would appear as if then [uhm] the information might be used like anyhow. But then that patient is. . . who was living and has relation, I wouldn’t want to then later see maybe an article published coding my name as one of the participants. (HCW IDI 03)

## Discussion

Deferred consent provides a means of addressing the practical difficulties to obtaining prospective informed consent in an emergency situation to enable medical research in emergency patients (Woolfall, Firth, Gamble, & Young, 2013). There was general agreement by all stakeholders interviewed on the acceptability of deferred consent in ECR, particularly because of the potential therapeutic benefits, which could accrue from taking emergency action to improve clinical outcomes. However, key factors influencing the acceptability of deferred consent in ECR depended on the nature or type of study (observational or interventional) with a preference given to minimally invasive observational research owing to the level and nature of risk and the severity or seriousness of the disease. These findings are consistent with other literature on the acceptability of deferred consent in ECR (Furyk et al., 2017; Maitland et al., 2011). Specifically, our findings mirror the findings of Gobat et al. (2015), who reported that stakeholders (patients, their proxy decision-maker, clinicians, and regulators) see deferred consent for ECR as justified for minimal-risk studies with a preference for observational as opposed to interventional research. The application of deferred consent during pandemics was acceptable. However, there were some specific barriers to deferred consent as perceived by our stakeholders.

### Legal Framework

The Malawian Constitution enshrines the right to autonomy, and the current regulatory guidelines present researchers considering the use of deferred consent with legal limits. It is only recently that the European countries have moved to allow the use of deferred consent in research. The United Kingdom and the European Union recently changed legislation to allow protocols using this method (The Medicines for Human Use (Clinical Trials) and Blood Safety and Quality (Amendment) Regulations [2008] and European Commission (European Clinical Trial Directive 2001/20/EC) [2011]). The United States have explicit provision for ECR and Final Rule developed by the U.S. DHHS, which allows for research to be performed without informed consent in emergency situations (U.S. Food & Drug Administration, 2011). Strengthening governance structures and regulatory capacity, including training REC members on deferred consent is essential. Others have also advocated for co-opting specific professional expertise in clinical, ethical, and psychosocial aspects (le-Roux-Kemp, 2014).

### Considerations of Autonomy and Problematizing Proxies

There are different conceptualizations of autonomy. Kushe and Singer (2013) note that while the West emphasizes individual autonomy, non-Western cultures place greater emphasis on cultural, communal, or family autonomy. In these communities, “family-centered” decision-making style (Blackhall, Murphy, Frank, Michael, & Azen, 1995) may determine decisions to participate in research with less emphasis on the primacy of the individual (National Health and Medical Research Council, the Australian Research Council and Universities Australia, 2007). Our findings echo these observations, although REC member, community member, and HCW indicated considerable uncertainty regarding who should give proxy consent. Some community members voiced their view that doctors or researchers should proceed without obtaining proxy consent because they would be acting in the best interests of the patient. However, REC members and HCW advocated for the use of close relatives as proxies to minimize the risk of guardians and family members blaming doctors or researchers of causing harm in the event that a patient dies. Considering the use of the deceased’s data, responses varied. Some promoted this to prevent bias in study results and others argued that additional express permission from surrogate decision-makers should be sought. This echoes Furyk et al.’s work (2017) who reported similar disagreement among parents of children who had participated in deferred consent research. Furthermore, evidence from the Fluid Expansion as Supportive Therapy (FEAST) trial reveals that deferred consent from proxies, particularly, after the death of the patient was not encouraged, as it appeared insensitive, inhumane, and inappropriate (Molyneux et al., 2013). There remain potential sociocultural challenges of not fully informing caregivers or legal guardians, which might result in erosion of trust in future research (Gamble et al., 2012).

We suggest that appropriate proxy consent guidelines for deferred consent research should be developed, which could minimize delays, reduce the possibility of blame, and formalize the ethical position for researchers’ use of deceased patients’ data.

## Best Practices

Community engagement is a key part of research planning and should take part before ethics submission. This was raised as a prerequisite to acceptable use of deferred consent. However, for ECR, it is difficult to identify and engage with potential research participants due to the unpredictable and relatively infrequent occurrence of acute disease. Community consultation and sensitization activities should, therefore, be focused on explaining what ECR entails and how deferred consent works and the various approaches used to obtain consent while still respecting the participant’s autonomy in research (Maitland et al., 2011), which might mitigate some of the challenges when researchers consider conducting emergency or critical care research. However, broad consultation is still required. Nyirenda et al. (2018) propose a community engagement model of collaboration, consultation, and communication. They argue that researchers should move beyond “the use of community engagement for ‘instrumental’ purposes,” and instead “the ‘intrinsic’ value of showing respect and ensuring inclusive participation of community partners in research design.” We agree with the suggestions that Nyirenda et al. (2018) put forward including the use of the following: (a) guidelines and checklists for planning community engagement, (b) training in community engagement at institutional level, (c) attention to community engagement within national policy on health research, and (d) explicit demands for descriptions of community engagement plans during REC reviews.

## Research Agenda

Our approach to advancing the use of deferred consent in Malawi will require building a broader consensus from all stakeholders on the procedures and processes for the implementation of ECR. This conversation (that hopefully results in normative guidelines) may consider the norms and standards applied in other jurisdictions, including those outlined above. As part of this conversation, further consultation with a wider range of stakeholders and in other settings could build on our findings, including deliberative research with communities that involves provision of information about deferred consent and frameworks in other countries and discussion of potential processes. Research on the perspectives of patients and guardians who have experienced critical research care directly would be particularly important (Rebers, Aaronson, van Leeuwen, & Schmidt, 2016) and offer the research ethics community further insights into what works and what does not.

## Educational Implications

Following on from the suggestions provided above, it is imperative that researchers and REC members continue to be mindful of the legislative guidelines on consent in research in Malawi. In addition, REC members should undertake short, continuous professional development training courses to increase understanding that will enable members to provide specific guidance and regulatory oversight to investigators regarding the use of deferred consent in ERC in Malawi.

## Conclusion

Deferred consent has not been considered for ECR in Malawi and, at present, there are no definitive ethical guidelines on the use of deferred consent in research. Our study was based on a small sample (*n* = 23), but provides insights into the acceptability of deferred consent for ECR in Malawi. Although deferred consent in ECR can be potentially transformative for individual and population health, concerns around malpractice must be robustly addressed through stringent oversight, tailored training, and broad community engagement. Particular consideration should be given to ECR studies that provide scientific value and have the potential to improve medical care and individual and/or population health. The research must include community consultation and public disclosure, and the regulatory environment must provide robust local oversight.

## Supplemental Material

Appendix_1-_Topic_guide_for_Key_Informant_Interviews_Research_ethics_participants – Supplemental material for A Formative Qualitative Study on the Acceptability of Deferred Consent in Adult Emergency Care Research in Malawi Supplemental material, Appendix_1-_Topic_guide_for_Key_Informant_Interviews_Research_ethics_participants for A Formative Qualitative Study on the Acceptability of Deferred Consent in Adult Emergency Care Research in Malawi by Lucinda Manda-Taylor, Fanuel Meckson Bickton, Kate Gooding and Jamie Rylance in Journal of Empirical Research on Human Research Ethics

Appendix_2-_Topic_guide_for_in-depth_interviews_Healthcare_workers – Supplemental material for A Formative Qualitative Study on the Acceptability of Deferred Consent in Adult Emergency Care Research in Malawi Supplemental material, Appendix_2-_Topic_guide_for_in-depth_interviews_Healthcare_workers for A Formative Qualitative Study on the Acceptability of Deferred Consent in Adult Emergency Care Research in Malawi by Lucinda Manda-Taylor, Fanuel Meckson Bickton, Kate Gooding and Jamie Rylance in Journal of Empirical Research on Human Research Ethics

Appendix_3-_Topic_guide_for_Focus_Group_Discussions_Community_participants – Supplemental material for A Formative Qualitative Study on the Acceptability of Deferred Consent in Adult Emergency Care Research in Malawi Supplemental material, Appendix_3-_Topic_guide_for_Focus_Group_Discussions_Community_participants for A Formative Qualitative Study on the Acceptability of Deferred Consent in Adult Emergency Care Research in Malawi by Lucinda Manda-Taylor, Fanuel Meckson Bickton, Kate Gooding and Jamie Rylance in Journal of Empirical Research on Human Research Ethics

## References

[bibr1-1556264619865149] Blackhall L. J. Murphy S. T. Frank G. Michael V. Azen S. (1995). Ethnicity and attitudes towards patient autonomy. Journal of American Medical Association, 274, 820-825. doi:10.1001/jama.1995.035301000 7650806

[bibr2-1556264619865149] Constitution of the Republic of Malawi. (1994). Chapter IV, section 19, point 5. Retrieved from http://www.sdnp.org.mw/constitut/chapter4.html

[bibr3-1556264619865149] Council for International Organizations of Medical Sciences. (2016). International ethical guidelines for biomedical research involving human subjects (4th ed.). Geneva, Switzerland: Author. Available from http://www.cioms.ch/14983848

[bibr4-1556264619865149] Department of Health. (2006). Guidelines for good practice in the conduct of clinical trials with human participants in South Africa (2nd ed.). Pretoria, South Africa: Author.

[bibr5-1556264619865149] European Commission. (2012). European Clinical Trial Directive 2001/20/EC of the European Parliament and of the Council of 4 April 2011. Retrieved from https://ec.europa.eu/health/human-use/clinical-trials/directive_en

[bibr6-1556264619865149] Furyk J. McBain-Rigg K. Renison B. Watt K. Franklin R. Emeto T. . . . Dalziel S. (2018). A comprehensive systematic review of stakeholder attitudes to alternatives to prospective informed consent in paediatric acute care research. BMC Medical Ethics, 19, Article 89. doi:10.1186/s12910-018-0327-9 PMC6245534 30453948

[bibr7-1556264619865149] Furyk J. McBain-Rigg K. Watt K. Emeto T. I. Franklin R. C. Franklin D. . . . Ray R. (2017). Qualitative evaluation of a deferred consent process in pediatric emergency research: A PREDICT study. BMJ Open, 7, e0181562. doi:10.136/bmjopen-2017-018562 PMC5695338 29146655

[bibr8-1556264619865149] Gamble C. Nadel S. Snape D. McKay A. Hickey H. Williamson P. . . . Young B. (2012). What parents of children who have received emergency care think about deferring consent in randomized trials of emergency treatments: Postal survey. PLoS ONE, 7, e35982. doi:10.1371/journal.pone.0035982 22586456 PMC3346812

[bibr9-1556264619865149] Gobat N. H. Gal M. Francis N. A. Hood K. Watkins A. Turner J. . . . Nichol A. (2015). Key stakeholder perceptions in acute illness research: A rapid, systematic review to inform EPI/pandemic research preparedness. Trials, 16, Article 591. doi:10.1186/s13063-015-1110-6 PMC4693405 26715077

[bibr10-1556264619865149] Gupta U. C. (2013). Informed consent in clinical research: Revisiting a few concepts and areas. Perspectives in Clinical Research, 4, 26-32. doi:10.4103/2229-3485.106373 23533976 PMC3601699

[bibr11-1556264619865149] Jansen T. C. Kompanje E. J. O. Druml C. Menon D. K. Wiederman C. J. Bakker J. (2007). Deferred consent in emergency intensive care research: What if the patient dies early? Use the data or not? Intensive Care Medicine, 33, 894-900. doi:10.1007/s00134-007-0580-8 17342513 PMC1964781

[bibr12-1556264619865149] Kushe H. Singer P. (2013). A companion to bioethics. Oxford, UK: Oxford University Press.

[bibr13-1556264619865149] le-Roux-Kemp A. (2014). Deferred consent in emergency care research: A comparative perspective of South African regulations. The Journal of Philosophy, Science & Law: RCR Special Issue, 14. Retrieved from http://www.researchgate.net/publication/259977302

[bibr14-1556264619865149] Maitland K. Molyneux S. Boga M. Kigali S. Lang T. (2011). Use of deferred consent for severely ill-children in a multi-centre phase III trial. Trials, 12, 90. Retrieved from http://www.trialsjournal.com/content/12/1/90 21453454 10.1186/1745-6215-12-90 PMC3077324

[bibr15-1556264619865149] Mason S. A. Allmark P. J. (2000). Obtaining informed consent to neonatal randomized controlled trials: Interviews with parents and clinicians in the Euricon study. The Lancet, 356, 2045-2057. 10.1016/s0140-6736(00)03401-2 11145490

[bibr16-1556264619865149] The Medicines for Human Use (Clinical Trials) and Blood Safety and Quality (Amendment) Regulations. (2008). Available from www.legislation.gov.uk

[bibr17-1556264619865149] Mentzelopoulos S. D. Mantzanas M. van Belle G. Nichol G. (2015). Evolution of European Union legislation on emergency research. Resuscitation, 91, 84-91. doi:10.1016/j.resuscitation.2015.03.006 25796997

[bibr18-1556264619865149] Molyneux S. Njue M. Boga M. Akello L. Olupot-Olupot P. Engoru C. . . . Maitland K. (2013). “The words will pass with the blowing wind”: Staff and parent views of the deferred consent process, with poor assent, used in an emergency fluids trial in two African hospitals. PLoS ONE, 8(2), e54894. doi:10.1371/journal.pone.0054894 23408950 PMC3569446

[bibr19-1556264619865149] National Commission for Science and Technology. (2011). The framework of guidelines for research in the social sciences and humanities in Malawi. Retrieved from https://www.ncst.mw/wp-content/uploads/2014/03/NATIONAL-FRAMEWORK-OF-GUIDELINES-IN-SSH.pdf

[bibr20-1556264619865149] National Health and Medical Research Council, the Australian Research Council and Universities Australia (2007). National statement on ethical conduct in human research in 2007—Updated 2018. Canberra, Australia: Commonwealth of Australia. Retrieved from https://www.nhmrc.gov.au/about-us/publications/national-statement-ethical-conduct-human-research-2007-updated-2018#toc__296

[bibr21-1556264619865149] Nowell L. S. Norris J. M. White D. E. Moules N. J. (2017). Thematic analysis: Striving to meet the trustworthiness criteria. International Journal of Qualitative Methods, 16, 1-3. doi:10.1177/16094069/7733847

[bibr22-1556264619865149] Nyirenda D. Gooding K. Sambakunsi R. Seyama L. Mfutso-Bengo J. M. Manda-Taylor L. . . . Parker M. (2018). Strengthening ethical community engagement in contemporary Malawi. Wellcome Open Research, 3, Article 115. doi:10.12688/wellcomeopenres.14793.1 PMC6259484 30542663

[bibr23-1556264619865149] Rebers S. Aaronson N. K. van Leeuwen F. E. Schmidt M. K. (2016). Exceptions to the rule of informed consent for research with an intervention. BMC Medical Ethics, 17, Article 9. doi:10.1186/s12910-016-0092-6 PMC4744424 26852412

[bibr24-1556264619865149] Toto N. T. (2016). Regulation of research with children in Malawi and challenges that arise when doing research with children. In Research with children: Ethical processes and challenges around the world. Nuffield Council of Bioethics. Retrieved from http://nuffieldbioethics.org/wp-content/uploads/Research-with-children-ethical-challenges-around-the-world_web.pdf

[bibr25-1556264619865149] Tu J. V. Willison D. J. Silver F. L. Fang J. Richards J. A. Laupacis A. Kapral M. K. (2004). Impracticality of informed consent in the registry of the Canadian stroke network. The New England Journal of Medicine, 350, 1414-1421. 15070791 10.1056/NEJMsa031697

[bibr26-1556264619865149] U.S. Department of Health and Human Services. (2013). Guidance for institutional review boards, clinical investigators, and sponsors: Exception from informed consent requirements for emergency research. Retrieved from https://www.fda.gov/downloads/RegulatoryInformation Guidances/UCM249673.pdf

[bibr27-1556264619865149] U.S. Food & Drug Administration. (2011). Guidance for Institutional Review Boards, Clinical Investigators, and Sponsors: Exception from informed consent requirements for emergency research. Retrieved from http://www.fda.gov/downloads/RegulatoryInformation/Guidances/UCM249673.pdf

[bibr28-1556264619865149] Universal Declaration on Bioethics and Human Rights (n.d.). Retrieved from http://unesdoc.unesco.org/images/0014001461/146180E.pdf

[bibr29-1556264619865149] Woolfall K. Firth L. Gamble C. Young B. (2013). How experience makes a difference: Practitioners’ views on the use of deferred consent paediatric and neonatal emergency care trials. BMC Medical Ethics, 14. Retrieved from http://www.biomedcentral.com/1472-6939/14/45 10.1186/1472-6939-14-45 PMC4228267 24195717

