# Supplementary material for: A Formative Qualitative Study on the Acceptability of Deferred Consent in Adult Emergency Care Research in Malawi
Source: J Empir Res Hum Res Ethics. 2019 Aug 8;14(4):318–27. doi: 10.1177/1556264619865149 (PMC13029355; doi:10.1177/1556264619865149)
Supplement: Appendix_1-_Topic_guide_for_Key_Informant_Interviews_Research_ethics_participants – Supplemental material for A Formative Qualitative Study on the Acceptability of Deferred Consent in Adult Emergency Care Research in Malawi [file Appendix_1-_Topic_guide_for_Key_Informant_Interviews_Research_ethics_participants.doc]

**Appendix 1: Topic guide for Key Informant Interviews (Research ethics participants)**

This guide may be adapted during the research in response to emerging issues.

## **Definition and explanation [read out verbatim]**

*“Traditionally, before people take part in research studies, they must give their consent to do so. They must understand the choice they are making, and choose without pressure. In emergency care settings, people may not have enough time, or they may be incapacitated, meaning that they cannot give informed consent.*

*In these circumstances, “deferred consent” has sometimes been used. Researchers start to include patients within their study, and aim to obtain full informed consent (from the patient) or proxy consent (from, for example, a relative) as soon as possible.”*

## **Background experience**

1. Your experience (if any) of dealing with trials with deferred consent
   - In what capacity were you involved?
   - How long have you been involved in this way?
   - What type of trial was it / were they?
2. What are your reflections on these trials with respect to deferred consent?

**Further discussion [open questions to participants]**

1. Exploring deferred consent in Malawi in general terms *[Open questions, which can be further probed]*
   - What do you see as the potential benefits of deferred consent?
   - What do you see as the risks or disadvantages of deferred consent?
   - Are there any practical issues that might make deferred consent harder or easier?
   - What do you see as the ethical issues around deferred consent?
   - How would you feel about supporting a study that uses deferred consent? What would your concerns be/what would make it OK to support the study?
   - When do you think deferred consent might be appropriate?
   - What could doctors or researchers do so that deferred consent is OK?
2. Does the validity depend on the trial type?
   - *[Prompt: compare an intervention trial where new medications are being given compared with observational study where only blood samples and clinical data are being taken]*
3. Does any person need to give consent on behalf of the participant where deferred consent is employed?
   - An independent doctor?
     - *[Prompt: What happens to the status of deferred consent when a relative arrives at the hospital?]*
   - A relative or caregiver?
     - To what degree should their wishes be respected?
     - *[Prompt: be specific about how researchers would know who was “acceptable”]*
     - *[Prompt: what happens if more than one relatives do not agree?]*
4. What if nobody arrives to give proxy consent, and the patient is still unable to consent?
   - Must consent from somebody always be obtained e.g. if a patient has died?
     - *[Prompt: Is it fair to ask relatives to proxy-consent after a patient has died?]*
     - *[Prompt: How long is deferred consent valid?]*
5. Are you aware of any local / national guidance on the issue?
   - Is it allowable within the current REC constitution and the law?

## **Logical extensions of the use / non-use of deferred consent**

1. Selection bias?
   - *[Explanation: if we used standard consent, only patients who had mild disease could be studied. This would mean that we still did not know what would happen in severe disease.]*
   - Does this matter?
2. Is there a wider argument for societal good?
   - *[Explanation: if we do not know the best way to treat patients, what do you think of deferred consent for studies which are responsive to the health needs and priorities of the specific vulnerable population or community, or where the population or community stand to benefit from the knowledge, practices or interventions that may result from the research.]*
3. Does the risk of the study treatment or procedures make any difference to whether deferred consent could be used?

## **Implementing deferred consent**

1. What checks and balances need to be in place to make sure deferred consent is conducted correctly?
   1. Within a study?
   2. Within the host research institution?
   3. Within the hospital / place of care?
   4. At the REC?
   5. At the governmental / legislative level?
